# Effect of manganese preconditioning and replacing inorganic manganese with organic manganese on performance of male broiler chicks

**DOI:** 10.3382/ps/pey564

**Published:** 2018-12-22

**Authors:** S Mwangi, J Timmons, T Ao, M Paul, L Macalintal, A Pescatore, A Cantor, K A Dawson

**Affiliations:** 1 Food Science and Technology Program, Department of Agriculture, Food and Resource Sciences, University of Maryland Eastern Shore, Princess Anne, Maryland 21853; 2 Alltech-University of Kentucky Nutrition Research Alliance, Lexington, Kentucky 40546

**Keywords:** preconditioning, excreta, post-hatch, organic, inorganic

## Abstract

The effects of manganese (Mn) preconditioning, 96 h post-hatch followed by the replacement of inorganic Mn with different levels of organic Mn (5 to 21 D), on growth, tissue excreta Mn content, gene expression, and enzyme activity were evaluated. A total of 420 day-old male Cobb 500 broilers were divided into 2 groups. One group was fed a corn–soybean meal basal diet containing 17 mg of Mn/kg (preconditioning diet, MnPD); the second group was fed the non-preconditioning diet (NPCD), which was the MnPD supplemented with 60 mg of Mn/kg from manganese sulfate (MnSO_4_). On day 5, each group was divided into 5 subgroups and were randomly assigned to dietary treatments consisting of MnPD alone or MnPD supplemented with 12 or 60 mg Mn/kg Mn as MnSO_4_ or Mn proteinate (6 replicate cages of 6 birds). Broiler chicks that were fed the MnPD had lower (*P* ≤ 0.05) body weight gain (BWG) and G:F ratio when compared to those that were fed the NPCD for 4 D. Birds that were fed MnPD (1 to 4 D) and switched to MnPD supplemented with 60 mg/kg Mn (5 to 21 D) had lower (*P* ≤ 0.05) BWG compared to those that were fed NPCD (1 to 4 D) and switched to MnPD supplemented with 60 mg/kg Mn for 21 D. Excreta, tibia ash, liver, and heart Mn levels were increased (*P* ≤ 0.05) by supplemental Mn. The expression of jejunum divalent metal transporter-1 mRNA levels, as well as activities of plasma total super oxide dismutase and liver alanine transaminase, was not affected by MnPD or Mn source and levels. These results confirmed that feeding marginally deficient Mn diets to broiler chicks post-hatch does affect growth rate and tissue Mn concentration.

## INTRODUCTION

Manganese (Mn) is an essential trace mineral in animal nutrition. It is involved in the activation of metalloenzymes that contribute to the metabolism of carbohydrates, lipids, and amino acids (Kies, 1994; Crowley et al., 2000; Suttle, 2010). It is also an important component of the Mn superoxide dismutase (Mn-SOD) that protects cells from oxidative stress (Luo et al., 1992; Li et al., 2011). The deficiency of Mn is associated with structural and physiological disorders, which include a reduced oxidant defense system, skeletal and cartilage malformation, and impaired reproductive function (Luo et al., 1992; Tuormaa, 1996). The National Research Council (NRC, 1994) recommended level of Mn for optimal growth of broiler chickens is 60 mg/kg. However, the commercial broiler industry typically formulates diets to contain higher amounts of trace minerals including Mn mostly from inorganic sources (Leeson and Caston, 2008).

Organic trace minerals have been shown to be more bioavailable than inorganic minerals (Wedekind et al., 1992; Cao et al., 2000; Burrell et al., 2004; Ao et al., 2006; Ji et al., 2006a). The improved bioavailability of organic trace minerals is hypothesized to be due to minimum interference from dietary antagonisms, such as phytic acid, and maintenance of their structural integrity through the digestive tract that allows them to reach absorption sites in the small intestine (Ashmead, 1993). Due to the increased bioavailability, organic trace minerals can be supplemented in broiler diets below the NRC recommendation without negatively impacting broiler performance. As a result, mineral excretion is reduced, which may be beneficial to the environment as there is a concern with excess minerals in poultry manure applied as fertilizer to crop fields (Jackson et al., 2003; Leeson, 2003; Ao et al., 2006; Bao et al., 2007; Lei et al., 2007; Nollet et al., 2008; Aksu et al., 2011).

Recent research has also shown that the manipulation of broiler diets during the critical stage of development post-hatch can affect their development later in life (Sklan and Noy, 2000; Willemsen et al., 2010). Broiler chicks provided feed immediately post-hatch were reported to have improved growth and metabolic rate, which was reflected by their ability to maintain body temperature when exposed to cold compared to those subjected to delayed feeding (Van den et al., 2010). Zhan et al. (2007) reported that early feed restriction in broilers for the first 3 wk post-hatch had a lasting impact on metabolic programming, which led to obesity in adult broilers. Additionally, the development of the gastrointestinal tract and maturation of enzyme secretion were shown to be affected by feed restriction in hatchlings (Uni et al., 1999). Results obtained by Yan et al. (2005) indicated that feeding broiler chicks a diet low in phosphorus (P) and calcium (Ca) from hatch to 18 D of age triggered adaptive responses that were demonstrated by increased absorption of P and Ca when compared to birds that were fed the recommended P and Ca levels. Results reported by Mwangi et al. (2016) also indicated that Zn imprinting affected broiler performance and body Zn stores of broiler chicks raised to 21 D.

Tissue Mn concentration in poultry is tightly regulated by adaptive changes when various dietary Mn levels are fed. When increased dietary Mn levels are fed to broiler chicks, there is reduced Mn absorption in the gastrointestinal tract and increased biliary and pancreatic excretion (Roth and Garrick, 2003; Suttle, 2010). However, it is not clear whether feeding broiler chicks a Mn-deficient diet for the first 96 h post-hatch and replacing inorganic Mn with different levels of proteinate Mn can promote any adaptive responses later in life. The objective of this study was to determine the effect of feeding a Mn-restricted diet (preconditioning diet) using a practical corn–soy diet with zero supplemental Mn to broiler chicks for the first 96 h post-hatch and later (5 to 21 D) feeding a basal diet supplemented with different levels and sources of Mn on broiler performance and tissue Mn content.

## MATERIAL AND METHODS

### Chicks and Housing

In this study, 420 1-day-old (Cobb 500) male broiler chicks were used. Six replicates of seven chicks per treatment were randomly allotted by weight to mesh wired floored pullet cages (61 cm × 51 cm × 36 cm) containing 2 adjustable nipple drinkers and 1 plastic feeder. Room temperature was maintained at 31°C for the first week and then decreased and maintained at 27°C to the end of the trial. Feed and water were offered ad libitum. A light schedule of 22 h light and 2 h dark was used throughout the trial. The analyzed Mn in the water was below detectable levels. All experimental procedures were approved by the University of Kentucky Institutional Animal Care and Use Committee.

### Experimental Design and Diets

In this study, a randomized complete block design was used. Blocking was based on cage location within the room, and each cage was considered an experimental unit. A corn and soybean meal basal diet (Table 1) without phytase was formulated to meet all NRC broiler nutrient requirements except Mn (NRC, 1994). During the Mn preconditioning period (MnPP) (0 to 4 D), chicks were either fed the basal diet analyzed to contain 17 mg of Mn/kg (Mn preconditioning diet-MnPD) or basal diet supplemented with 60 mg Mn/kg of feed grade MnSO_4_–H_2_O (non-preconditioning diet—NPCD). At the end of MnPP, (day 5), 6 replicate cages (6 chicks per cage) from each group were randomly assigned to basal diet alone (MnPD) or basal diet supplemented with 12 or 60 mg/kg Mn as MnSO_4_–H_2_O or Bioplex Mn (Alltech Inc. Nicholasville, KY; Table 2).

**Table 1.** Composition of the basal diet fed to broiler chicks to evaluate the effect of dietary imprinting.

Ingredient:	%
Corn	59.17
SBM, dehulled (48% cp)	33.70
Corn oil	3.00
Dical (technical grade)	0.80
Dical (feed grade)	0.81
Calcium carbonate (technical grade)	1.26
Salt, iodized	0.45
DL-methionine	0.21
L-lysine	0.10
Vitamin premix^1^ (no mineral)	0.25
Mineral premix^2^ (no Mn)	0.25
Total	100.00
Calculated(analyzed)nutrient	
ME, kcal/kg	3080
CP, %	21
Ca^3^, %	1.00 (1.2)
Available P^3^, %	0.45(0.44)
Lysine, %	1.24
Methionine + Cystein, %	0.90
Manganese (mg/kg)	17

^1^Vitamin premix per kilogram of diet: 11,025 I.U. vitamin A; 3,528 I.U. vitamin D; 333 I.U. vitamin E; 0.91 mg vitamin K; 2 mg thiamin; 8 mg riboflavin; 55 mg niacin; 18 mg Ca pantothenate; 5 mg vitamin B.

^2^Mineral premix per kilogram of diet: 0.264 mg Se as Na_2_SeO_3_; 12.80 mg Cu as CuSO_4_; 0.24 mg I as KIO_3_; 106.67 mg Fe as FeSO_4_.H_2_O; 22.22 mg Zn as ZnO.

^3^Determined by the analysis of duplicate samples.

**Table 2.** Calculated and assayed dietary manganese (Mn) levels of broiler diets fed to broiler chicks from 5 to 21 D.

Mn source	Calculated Mn content (mg/kg)	Analyzed Mn content (mg/kg)^1^
Basal diet	0	16.65
MnSO_4_–H_2_O	12	32.49
	60	84.31
Bioplex Mn	12	30.35
	60	78.41

^1^Analyzed Mn values are based on chemical analysis of triplicate samples of each diet.

### Broiler Performance, Sample Collection, and Mn Analysis

Body weight gain (BWG) and feed intake (FI) for each cage were recorded on days 1, 5, 14, and 21. Gain-to-feed ratio (G:F) of each cage was determined by dividing total BWG by total FI.

Blood, liver, heart, and jejunum samples were collected from one bird of average weight from each cage (6 birds per treatment) on 5 and 21 D of age. The birds were euthanized using argon gas immediately after blood samples were obtained through cardiac puncture. The blood was transferred into heparinized tubes and centrifuged at 1000 × *g* for 15 min at 4°C. The plasma was frozen at −20°C for later analysis of total superoxide dismutase (T-SOD). Liver samples were removed, rinsed in cold phosphate buffer saline at a pH of 7.4, and frozen in liquid nitrogen. Samples were later stored at −20°C for metabolic enzymes analysis of alanine transaminase (ALT) and Mn concentration.

A segment of the jejunum (2.5 cm) near Meckel's diverticulum was excised, rinsed with cold phosphate buffer saline (0.1 M, pH 7.4), placed in plastic cryogenic vial, and snap-frozen in liquid nitrogen. Samples were later transferred to −80°C for the analysis of mRNA expression level of divalent metal transfer 1 (DMT1) using real-time polymerase chain reaction (RT-PCR).

Right tibia bones from 2 birds per cage were pooled and boiled in deionized water for approximately 10 min to remove all of soft tissues. The bones were then dried at 60°C for 72 h, and fat extracted in petroleum ether for 3 D. The defatted tibia bones were dried for 12 h at 105°C and cooled to room temperature in desiccators. The dried tibias were placed in a muffle furnace at 600°C overnight for ash determination and Mn analysis.

Excreta samples were collected on day 21 for 24 h from each replicate using collection trays. Non-excreta material was removed, and the excreta from each cage was pooled, weighed, and dried for 48 h at 105°C. Before mineral analysis, the dried excreta and feed samples were ground into fine particles using a coffee grinder and sealed in plastic bags. Samples were stored at 4°C until they were analyzed for Mn content.

### Acid Digestion and Mineral Determination

Before mineral analysis, approximately 1 g of the freeze-dried, homogenized liver and heart samples as well as tibia ash, excreta, and feed samples was microwave-digested with nitric acid for analysis of Mn using inductively coupled plasma emission spectroscopy (ICP-OES, axial 720 series) according to the method described in AOAC (1996).

### Liver and Plasma Metabolic Enzyme assay

Plasma T-SOD of each sample was measured in triplicates following the procedure described in superoxide dismutase assay kit (Cayman chemicals Co., Ann Arbor, MI). The alanine transferase (ALT) level in the liver homogenate was determined using enzyChrom ALT assay kit (Bioassy system, Hayward, CA).

### Total RNA Isolation

Total RNA isolation was prepared using Qiagen RNeasy Mini kit (Qiagen Inc., Valencia, CA) following the manufacturer's protocol. The quality and quantity of RNA were determined using Nanodrop ND-1000 spectrophotometry and Agilent 2100 bioanalyzer system following manufacturer's protocol (Agilent Technologies, Santa Clara, CA).

### cDNA synthesis

Complementary DNA (cDNA) was synthesized from 0.5 μg of total RNA from each sample using the high-capacity cDNA reverse transcription kit (Applied Biosystems, Foster city, CA). All reactions were conducted in a 20 μl volume containing 10 μl of total RNA (50 ng/μl) and 10 μl master mix (2 μl 10× RT buffer, 0.8 μl 25× dNTP, 1 μl reverse transcriptase, and 4.2 μl nuclease free water). The 3 thermo-cycles consisted of an initial primer extension for 10 min at 25°C, 120 min at 37°C cDNA synthesis, and inactivation of RT transcriptase for 5 min at 85°C. The cDNA products were stored at −20°C.

### Real-time Polymerase Chain Reaction

RT-PCR analysis was carried out in triplicate using Taqman advanced master mix using the 7,500 RT-PCR system (Applied biosystem Foster City, CA). DMT1-specific primers (GenBank accession number EF635922) and β-actin (GenBank accession number L08165) were purchased from Thermo Fisher Scientific (Grand Island, NY). The sequences of primer for DMT1 were as follows: forward 5′-AGCCGTTCACCACTTATTTCG-3′, reverse 5′-GGTCCAAATAGGCGATGCTC-3′, and primers pair for β -actin—forward 5′-GAGAAATTGTGCGTGACATCA-3′, reverse 5′-CCTGAACCTCTCATTGCCA-3′. A 5 μl cDNA sample was diluted (1:25) with DNase-free water and mixed with a working reagent that contained 10 μl Taqman enzyme, 1 μl specific PCR primers, and 4 μl nuclease free water. The RT-PCR reaction conditions consisted of 50°C for 2 min; 95°C for 20 s; 40 cycles of 95°C for 3 s; and 60°C for 30 s. In each run, a non-template control was used to check for genomic contamination. Expression of DMT1 in each sample was normalized to β-actin and the relative quantification was expressed as a fold change of the target gene using 2^−ΔΔ^*^C^*^T^.

### Statistical Analysis

Single degree-of-freedom contrast was used to compare all supplemental levels with control to determine the effects of supplemental Mn (Li et al., 2011). Data for all variables excluding the control were further analyzed by 3-way analysis of variance (ANOVA) with the model including the main effect of Mn level, source, Mn preconditioning, and their interaction, using general linear model of Statistix V. 10 (Analytical Software, Tallahassee, FL). Tukey's HSD was used to determine means differences among treatments. Significant differences among treatments were determined at probability of *P* ≤ 0.05.

## RESULTS

### Growth Performance

At 5 D, broiler chicks that were fed the Mn preconditioning diet (MnPD) had significantly lower BWG and G:F when compared to those that were fed the NPCD (60 g/bird and 0.60 vs. 63 g/bird and 0.63, respectively). At 14 D, no significant difference was observed in performance parameters (*P* > 0.05). However, there was a trend towards improved BWG due to the interaction of Mn precondition period, Mn source, and level (*P* = 0.054) (Table 3). At 21 D, a significant 3-way interaction among the Mn preconditioning period, the Mn source, and the level was observed on BWG (*P* ≤ 0.05), but not on FI and G:F (Table 3). Birds that were fed the MnPD (1 to 4 D) and later (5 to 21 D) the diet supplemented with 60 mg/kg Mn had significantly lower BWG compared to the BWG of chicks that were fed the NPCD and MnPD (1 to 4 D) and subsequently (5 to 21 D) were fed the diet supplemented with 12 mg/kg Mn.

**Table 3.** Effects of feeding Mn preconditioning diet for the first 96 h post-hatch and replacing manganese sulfate with Bioplex Mn on growth performance of broiler chicks.

	Weight gain g/bird		Feed intake g/bird		Feed efficiency g/f	
Item	1 to 14 D	1 to 21 D	1 to 14 D	1 to 21 D	1 to 14 D	1 to 21 D
Mn preconditioning period (MnPP)						
MnPD^1^ (Mn preconditioning diet)	359	736	551	1,166	0.656	0.632
NPCD (non-preconditioning diet)	363	745	541	1,174	0.671	0.636
Mn source						
Basal diet^3^	363	734	550	1,166	0.730	0.632
Manganese sulfate^2^	364	744	546	1,168	0.670	0.637
Bioplex Mn^2^	359	738	545	1,171	0.660	0.631
Mn level (mg/kg)^2^						
12	366	753^a^	547	1,173	0.667	0.643
60	357	728^b^	544	1,167	0.660	0.625
Interaction of Mn level×MnPP						
12 mg/kg/MnPD	367	759^a^	550	1,174	0.668	0.647
12 mg/kg/NPCD	362	748^a^	544	1,172	0.667	0.640
60 mg/kg/MnPD	352	714^b^	545	1,157	0.645	0.617
60 mg/kg/NPCD	364	743^a,b^	542	1,176	0.676	0.633
*P*-values						
MnPP	0.325	0.562	0.612	0.526	0.212	0.665
Mn source	0.193	0.483	0.888	0.827	0.325	0.498
Mn level	0.092	0.004	0.667	0.643	0.536	0.065
MnPP × source	0.772	0.508	0.303	0.085	0.385	0.383
MnPP × level	0.055	0.020	0.828	0.416	0.178	0.243
Mn source × level	0.971	0.390	0.736	0.777	0.919	0.735
MnPP x source × level	0.055	0.008	0.198	0.447	0.766	0.167
SEM	2.582	4.698	4.110	5.966	0.006	0.004

^1^No supplemental Mn in the basal diet fed to chicks for 96 h.

^2^Data represent means of 24 replicates cages (n = 6).

^3^Data present means of 12 replicates cages (n = 6).

^a,b^Means within the same column within the same factor with any identical letters are not significantly different at *P* ≤ 0.05 by Tukey's HSD.

### Tissue Mineral Concentration

Tissue Mn concentrations are presented in Table 4. There was no significant interaction effect due to Mn precondition period, source, and level. However, Mn concentrations in tibia ash, heart, and liver were increased by supplemental Mn. Broiler chicks that were fed a diet supplemented with 60 mg/kg of Mn had higher (*P* ≤ 0.01) tissue Mn compared to those that were fed a diet supplemented with 12 mg/kg of Mn. Chicks maintained on MnPD to 21 D had lower (*P* ≤ 0.01) tibia ash Mn concentration when compared to those fed diets supplemented with either Bioplex Mn or MnSO_4_.

**Table 4.** Effects of Mn preconditioning diet and replacing MnSO_4_ with Bioplex Mn at different levels on tissues and excreta Mn from broiler chicks raised to 21 D.

Item	Tibia Ash Mn (mg/kg)	Tibia Ash (%)	Excreta Mn (mg/kg)	Liver Mn (mg/kg)	Heart Mn (mg/kg)
Mn preconditioning period (MnPP)					
MnPD^1^ (Mn preconditioning diet)	3.21	52.66	171.52	6.87	1.30
NPCD (non-preconditioning diet)	3.19	53.14	173.57	6.82	1.35
Mn source					
Basal diet^3^	1.71^b^	52.09	74.74^b^	5.84	1.12
Manganese sulfate^2^	3.64^a^	53.74	184.78^a^	7.25	1.34
Bioplex Mn^2^	3.39^a^	52.72	206.12^c^	6.84	1.38
Mn Level (mg/kg)^2^					
12	2.27^a^	52.58	117.92^a^	6.38^a^	1.17^a^
60	4.75^b^	53.76	281.00^b^	7.72^b^	1.54^b^
Interaction of Mn level × source					
12 mg/kg MnSO_4_.H_2_O	2.19	52.60	118.18^a^	6.75	1.14
12 mg/kg Bioplex Mn	2.35	52.59	117.66^a^	6.07	1.21
60 mg/kg MnSO_4_.H_2_O	5.09	55.03	264.70^b^	7.74	1.55
60 mg/kg Bioplex Mn	4.40	52.81	294.58^c^	7.72	1.54
*P*-values					
MnPP	0.760	0.336	0.815	0.621	0.837
Mn source	0.001	0.368	0.001	0.422	0.764
Mn level	0.001	0.235	0.001	0.004	0.001
MnPP × source	0.232	0.303	0.060	0.704	0.550
MnPP × level	0.840	0.200	0.495	0.619	0.857
Mn source × level	0.133	0.302	0.001	0.458	0.689
MnPP × source × level	0.874	0.114	0.580	0.181	0.756
SEM	0.210	0.129	11.820	0.210	0.05

^1^No supplemental Mn in the basal diet fed to broiler chicks for 96 h.

^2^Data represent the means of 24 replicates cages.

^3^Data represent the means of 12 replicates cages.

^a–c^Means with different superscript in the same column are significantly different at *P* ≤ 0.05 by Tukey's HSD.

At 21 D, excreta Mn was not influenced by preconditioning period, Mn source, and level or their interaction. However, excreta Mn content was affected (*P* ≤ 0.05) by the interaction of Mn source and level (Table 4). Excreta Mn concentrations from broiler chicks that were fed Mn supplemented diets were significantly high when compared with excreta Mn of the chicks that were fed Mn unsupplemented diet. Additionally, broiler chicks that were fed diet supplemented with 12 mg/kg Mn from both sources had lower excreta Mn concentration compared to excreta Mn concentration from chicks that were fed diets supplemented with 60 mg/kg Mn. However, excreta Mn from birds that were fed diet supplemented with 60 mg/kg of Bioplex Mn was higher than the excreta Mn concentration from chicks that were fed diets supplemented with 60 mg/kg Mn of MnSO_4_.

### Plasma and Liver Enzyme Activity

The activities of plasma T-SOD, and liver ALT activities determined on 5 and 21 D were not affected by Mn preconditioning period, source, and level or their interactions.

### Jejunum DMT1 mRNA Level Expression

The DMT1 mRNA expression levels in the jejunum were not affected by Mn preconditioning period, Mn source, and level or their interactions (*P* > 0.05) (Table 5). The results suggest that Mn preconditioning period as well as Mn source and levels did not have any impact on the expression of DMT1 mRNA in 5 and 21-day-old broiler chickens.

**Table 5.** Effects of Mn preconditions diet 96 h post-hatch and replacing of manganese sulfate with Bioplex Mn on plasma and liver enzyme activity and expression of jejunum divalent metal transfer 1 mRNA expression at 21 D.

Item	Plasma T-SOD (U/ml)	Liver ALT (U/L)^4^	DMT1
Mn preconditioning period (MnPP)			
MnPD^1^ (Mn preconditions diet)	52.86	898	1.03
NPCD (non-preconditioning diet)	54.26	932	0.98
Mn source			
Basal diet^3^	52.74	847	1.02
Manganese sulfate^2^	53.70	963	0.99
Bioplex Mn	53.80	905	1.02
Mn level (mg/kg)^2^			
12	54.99	918	1.01
60	52.45	950	0.99
Pooled SE			
	*P*-values		
MnPP	0.570	0.699	0.289
Mn source	0.680	0.590	0.778
Mn level	0.454	0.723	0.706
MnPP × source	0.666	0.167	0.370
MnPP × level	0.060	0.769	0.895
Mn Source × level	0.698	0.276	0.226
MnPP x source × level	0.3093	0.676	0.316
SEM	0.990	40.707	0.170

T-SOD – Total superoxide dismutase, ALT – alanine transaminase.

DMT1 – divalent metal transfer 1.

^1^No supplemental Mn in the basal diet fed to broiler chicks for 96 h.

^2^Data represent the means of 24 replicates cages.

^3^Data represent the means of 12 replicates cages.

^4^U/L: one unit catalyzes the formation of 1 μmole per liter of NAD per minute at 25°C.

## DISCUSSION

### Performance

It has been clearly established that dietary manipulation that includes trace minerals of broiler diets post-hatch can affect their development and performance later in life (Zulkifli et al., 1994; Zhan et al., 2007; Mwangi et al., 2016). The current NRC (1994) Mn recommended level for optimal performance in broiler chicken is 60 mg Mn/kg. However, in the commercial broiler industry the deficiency of Mn in broiler diets is not of concern due to the oversupply of trace minerals from inorganic sources (Leeson and Caston, 2008). In this experiment, feeding CSBD analyzed to contain 17 mg/kg Mn was intended to provide a marginally deficient level of Mn in broiler chicks.

Findings from this study showed that chicks that were fed Mn preconditioning diet had a significantly lower BWG and G:F compared to non-preconditioned chicks at 5 D of age. These results suggest that the current NRC (1994) recommended level for Mn (60 mg/kg) is required for maximum broiler performance. At 21 D of age, broiler chicks that were fed the MnPD (1 to 4 D) and later (5 to 21 D) were fed a diet supplemented with 60 mg/kg Mn had significantly lower BWG compared to those that were fed the NPCD and MnPD (1 to 4 d) and later (5 to 21 d) were fed a diet supplemented with 12 mg/kg Mn. (Table 3). Performance data observed in this study were in contrast from other studies that reported no effect on performance when broilers were fed diets supplemented with inorganic or organic Mn sources. (Bao et al., 2007; Luo et al., 2007; Nollet et al., 2007; Li et al., 2011; Brooks et al., 2012; Lu et al., 2006). The contrasting results from our study to those referenced above could be explained in part by difference in experimental design and the supplemental dietary levels. For instance, in a study conducted by Brooks et al. (2012) at the end of 7 D Mn depletion period, only broiler chicks with uniform body weight were used, while in our study broiler chicks were not weight matched after Mn preconditioning period (0 to 4 d). Although results from our study indicate that Mn preconditioning diet and supplemental Mn level affected BWG at 21 D, further studies are warranted to validate the reproducibility of these results taking into consideration that practical basal diet may have variable amount of trace mineral.

### Concentrations of Mn in Liver Tibia and Heart

In previous studies, tissue mineral accumulation has been used to determine body mineral utilization, storage, and bioavailability (Sunder et al., 2006; Wang et al., 2007; Suttle, 2010). A bioavailability study by Li et al. (2004) indicated that the heart and bone Mn content from broiler chicks were increased (*P* ≤ 0.05) as the dietary Mn level increased. However, no differences in the Mn bioavailability were detected between chicks that were fed a diet supplemented with inorganic or organic Mn sources. Similarly, Berta et al. (2004) reported that the supplementation of inorganic Mn (Mn oxide) and organic Mn (Mn fumarate) to a broiler diet at the same level did not have a significant effect on liver Mn content. However, liver Mn levels were linearly increased by feeding increased level of Mn.

The results of the current study indicated that there were no significant interactions due to Mn preconditioning period, source, and level on heart, liver, and tibia ash Mn concentration. However, increased supplemental Mn level to the basal diet resulted in increased heart, liver, and tibia ash Mn regardless of the Mn source. Similar to these results, Yan and Waldroup (2006) reported a linear response of tibia Mn due to increased Mn levels in broiler diets regardless of the Mn source (Mintrex Mn, reagent grade MnSO_4_ and reagent grade Mn monoxide). However, the rate of increased tibia Mn in response to increased dietary Mn was different among the 3 sources evaluated that resulted in a significant interaction between Mn source and level. Luo et al. (2007) indicated that Mn concentrations of heart and bone were more responsive to dietary Mn level. Conly et al. (2012) reported no significant difference between manganese sulfate (MnSO_4_) and tribasic Mn chloride based on a regression slope analysis of dietary Mn content on both tibia and liver Mn. Furthermore, the authors observed that the liver Mn content increased linearly (*P* ≤ 0.05) as the dietary Mn increased up to 60 ppm, but only numeric difference was detected in the liver Mn content from 60 to 130 ppm. Based on the results of the current study and the referenced studies, it can be concluded that the tissue Mn content is responsive to dietary Mn level, but it is not sensitive enough to detect differences among Mn sources.

The analysis of the data from the current study indicated that Mn preconditioning period, Mn source, and levels did not have any effect on percent tibia ash at 21 D. Yuan et al. (2011) reported that dietary replacement of inorganic Zn/Mn with organic Mintrex Zn/Mn had no effect on the dry weight, ash weight, and ash percentage of the metatarsus from broiler chicks raised to 21 D. However, a reduction of dietary Mintrex Zn/Mn resulted in a reduced metatarsus length. Similarly, results from a study by Sunder et al. (2011) evaluating the effect of graded levels of Mn supplemented at 100, 200, 400, 800, and 1,600 ppm indicated that there were no differences in tibia weight and percent tibia ash due to the graded Mn levels. In our study, it was expected that maintaining broiler chicks on Mn-deficient diet to 21 D could cause leg abnormalities; however, this problem was not observed.

### Expression of Jejunum DMT1

The role of DMT1 in Mn transportation across the microvillus into enterocytes has been described by Chua and Mogan (1997) and Trinder et al. (2000). DMT1 has also been suggested to be responsible for the transportation of other divalent transition metals including zinc, cobalt, cadmium, copper, nickel, lead, and iron, which are essential for physiological activities in the cells (Au et al., 2008). In the present study, Mn preconditioning period, source, and levels did not have any significant effect on the expression of DMT1 mRNA in the jejunum of the broiler chicks raised to 21 D. In contrast to our findings, Bai et al. (2008) reported that the expression of DMT1 mRNA levels in small intestine of broilers in Mn-supplemented group (analyzed to contain 117 mg Mn/kg) was lower (*P* < 0.01) compared to that of broilers that were fed the control diet analyzed to contain 18 mg Mn/kg. The effects of Mn source on DMT1 mRNA expression have also been reported (Bai et al., 2012). The results indicated that the expression of DMT1 mRNA levels was higher in ligated duodenum exposed to organic Mn compared to that of ligated duodenum exposed to inorganic Mn. The differences in experiment design, age of the birds, and the levels of dietary Mn might explain the discrepancies in results between our study and those reported above.

### Blood and Liver Metabolic Activity

Dietary Mn has been reported to affect the activation of Mn-SOD gene expression at transcriptional and translational levels (Gao et al., 2011). Mn-SOD is highly expressed in the liver, where the enzyme is required for inactivation of free radicals generated during regular metabolism activity (Whittaker, 2010; Li et al., 2011). Elevated levels of ALT, lactate dehydrogenase (LDH), creatinine kinase, glutamate-pyruvate transaminase (GTP) in the blood, and tissues can be an important indicator for tissue damage that can be caused by the increased level of reactive oxygen species or diseases (Shakoori et al., 1994; Grunkemeyer, 2010). In the present study, no significant difference in the plasma T-SOD and liver ALT activities were detected due to Mn preconditioning period, Mn source, and levels or their interactions. In agreement to our findings, Yuan et al. (2011) reported that there were no differences in the activities of serum T-SOD, CuZn-SOD, and MnSOD, and the liver metabolic enzyme activities of LDH, glutamic oxalacetic transaminase (GOT), and GTP among broilers that were fed a basal diet supplemented with 120 mg/kg Mn from sulfate salts compared to organic Zn/Mn supplemented diets at 100%, 80%, and 60% of the NRC recommendation. The authors concluded that the use of 80% supplementation of organic Zn/Mn may be enough to support and maintain LDH, GOT, and GTP enzyme activities. A study carried out by Aksu et al. (2010) demonstrated that the supplementation of organic Bioplex Zn, Cu, and Mn below the NRC recommendation levels to broiler diets had no significant effect on the plasma ALT, aspartate aminotransferase, and g-glutamyl transferase activities from broiler chickens raised to 42 D. Results from our study suggested that the Mn content of the basal diet was enough to support and maintain liver ALT and plasma T-SOD enzyme activities. These results could be explained in part by the fact that hepatocytes have the capacity to regenerate especially during liver damage, and up to 80% of the liver must be dysfunctional for any clinical diagnosis to be evident (Grunkemeyer, 2010).

### Excreta Mn Content

Excreta Mn content was influenced by Mn source and level (*P* ≤ 0.05). As expected, broiler chicks maintained on Mn preconditioning diet to 21 d had low excreta Mn content in comparison with the excreta Mn content from the chicks fed diets supplemented with 12 or 60 mg/kg Mn. The low levels of excreta Mn could be attributed to low Mn levels in the basal diet and the ability of broilers to sustain Mn homeostasis by increasing absorption and reducing excretion (Suttle, 2010).

The current results are similar to those obtained by Bao et al. (2007), which indicated that there was a linear increase (*P* < 0.05) in the excretion of evaluated organic trace minerals (Cu, Mn and Zn) in response to increased dietary minerals. Nollet et al. (2008) reported that substitution of 100% inorganic trace minerals with 100% organic trace minerals in broiler diets did not result in a significant reduction of the mineral excreta content except when low levels of organic minerals were fed. The authors suggested that the supplementation of high levels of chelated minerals does not translate to higher mineral retention. In contrast, Yuan et al. (2011) reported that 100% replacement of inorganic Zn/Mn in broiler diets with 100% organic Zn/Mn resulted in a significant reduction of Zn/Mn in the excreta. However, the authors suggested that supplementation of 80% of organic Zn/Mn in broiler diets resulted in less Zn/Mn in the excreta without compromising the growth performance of broiler chicks. Results from the current study indicated that low levels of inorganic and organic Mn could be supplemented in broiler diets without compromising performance.

In summary, results from this study clearly suggest that Mn preconditioning period did alter broiler chicks BWG at 21 D. Additionally, supplementation of 12 mg/kg of Mn from both sources was sufficient to support optimum broiler performance especially after preconditioning period; hence, it is apparent that dietary manipulation can be used to enhance efficiency utilization of trace minerals that could result in reduced mineral excretion. As such, additional studies are warranted to evaluate the effect of Mn preconditioning to 42 D and application of this practice in commercial broilers industry.
